# Correction: Pre-exposure prophylaxis (PrEP) awareness, use, and discontinuation among Lake Victoria fisherfolk in Uganda: A cross-sectional population-based study

**DOI:** 10.1371/journal.pgph.0004962

**Published:** 2025-07-21

**Authors:** The PLOS Global Public Health Staff

## Notice of republication

This article was republished on June 20, 2025, to correct errors in the text that were introduced during the typesetting process. The publisher apologizes for the errors. Please download this article again to view the correct version. The originally published, uncorrected article and the republished, corrected articles are provided here for reference.

## Supporting information

S1 File Originally published, uncorrected article. (PDF)

S2 File Republished, corrected article. (PDF)
