# Supplementary material for: Pre-exposure prophylaxis (PrEP) awareness, use, and discontinuation among Lake Victoria fisherfolk in Uganda: A cross-sectional population-based study
Source: PLOS Glob Public Health. 2025 May 9;5(5):e0003994. doi: 10.1371/journal.pgph.0003994 (PMC12063894; doi:10.1371/journal.pgph.0003994)
Supplement: S1 Table — (DOCX) [file pgph.0003994.s002.docx]

### **S2 Supplemental Table:** Individual-level correlates of PrEP ever use among 544 participants in a Lake Victoria Fishing community in southcentral Uganda in 2019 at substantial HIV risk/likely PrEP eligible stratified by gender.

| **Characteristics** | **Male participants (n=375)** | | | | | **Female participants (n=169)** | | | | |
| --- | --- | --- | --- | --- | --- | --- | --- | --- | --- | --- |
|  | **No.**  **Reporting/Total (%)** | **Unadjusted PR (95%CI)** | **P-value** | **Age-adjusted PR (95%CI)** | **P- value** | **No.**  **Reporting/Total (%)** | **Unadjusted PR (95% CI)** | **P-value** | **Age-adjusted PR (95%CI)** | **P- value** |
| **Age group (years)** |  |  |  |  |  |  |  |  |  |  |
| 15-19 | 4/24 (16.7) | 1.17 (0.41 – 3.33) | 0.774 | 1.17 (0.41 - 3.33) | 0.774 | 5/30 (16.7) | 0.46 (0.19 – 1.12) | 0.088 | 0.46 (0.19 - 1.12) | 0.088 |
| 20-24 | 11/77 (14.3) | Ref | Ref | Ref | Ref | 18/50 (36.0) | Ref | Ref | Ref | Ref |
| 25-29 | 16/80 (20.0) | 1.4 (0.69 – 2.82) | 0.347 | 1.40 (0.69 - 2.82) | 0.347 | 11/35 (31.4) | 0.87 (0.47 – 1.61) | 0.665 | 0.87 (0.47 - 1.61) | 0.665 |
| 30-34 | 14/73 (19.2) | 1.34 (0.65 – 2.77) | 0.425 | 1.34 (0.65 - 2.77) | 0.425 | 8/25 (32.0) | 0.89 (0.45 – 1.76) | 0.735 | 0.89 (0.45 - 1.76) | 0.735 |
| 35-39 | 14/67 (20.9) | 1.46 (0.71 – 3.00) | 0.300 | 1.46 (0.71 - 3.00) | 0.300 | 2/11 (18.2) | 0.51 (0.14 – 1.87) | 0.307 | 0.51 (0.14 - 1.87) | 0.307 |
| 40-44 | 9/34 (26.5) | 1.85 (0.85 – 4.06) | 0.123 | 1.85 (0.85 - 4.06) | 0.123 | 2/11 (18.2) | 0.51 (0.14 – 1.87) | 0.307 | 0.51 (0.14 - 1.87) | 0.307 |
| 45-49 | 3/20 (15.0) | 1.05 (0.32 – 3.42) | 0.935 | 1.05 (0.32 - 3.42) | 0.935 | 1/7 (14.3) | 0.40 (0.06 – 2.54) | 0.329 | 0.40 (0.06 - 2.54) | 0.329 |
| **Educational Status** |  |  |  |  |  |  |  |  |  |  |
| None | 4/17 (23.5) | 1.29 (0.53 – 3.15) | 0.580 | 1.19 (0.48 - 2.97) | 0.710 | 4/12 (33.3) | 1.19 (0.50 – 2.82) | 0.692 | 1.29 (0.51 - 3.23) | 0.588 |
| Primary | 51/279 (18.3) | Ref | Ref | Ref |  | 28/100 (28.0) | Ref | Ref | Ref | Ref |
| Secondary/Tertiary | 16/79 (20.3) | 1.11 (0.67 – 1.83) | 0.690 | 1.18 (0.70 - 1.97) | 0.536 | 15/57 (26.3) | 0.94 (0.55 – 1.61) | 0.821 | 0.87 (0.50 - 1.52) | 0.628 |
| **Primary occupation** |  |  |  |  |  |  |  |  |  |  |
| Other | 23/146 (15.8) | 0.73 (0.47 – 1.15) | 0.180 | 0.76 (0.48 - 1.23) | 0.266 | 33/125 (26.4) | 0.92 (0.51 – 1.69) | 0.797 | 0.95 (0.53 – 1.71) | 0.863 |
| Agrarian | 2/15 (13.3) | 0.62 (0.17 – 2.32) | 0.477 | 0.59 (0.16 - 2.14) | 0.465 | 4/9 (44.4) | 1.56 (0.63 – 3.83) | 0.337 | 1.74 (0.65 - 4.62) | 0.268 |
| Fishing | 46/214 (21.5) | Ref | Ref | Ref | Ref | - | - | - | - | - |
| Housework | - | - | - | - | - | 10/35 (28.6) | Ref | Ref | Ref | Ref |
| **Recent in-migrant*** |  |  |  |  |  |  |  |  |  |  |
| No | 55/306 (18.0) | Ref | Ref | Ref | Ref | 34/111 (30.6) | Ref | Ref | Ref | Ref |
| Yes | 16/69 (23.2) | 1.29 (0.79 – 2.11) | 0.311 | 1.44 (0.87 - 2.40) | 0.156 | 13/58 (22.4) | 0.73 (0.42 – 1.28) | 0.271 | 0.68 (0.39 - 1.18) | 0.170 |
| **Marital status** |  |  |  |  |  |  |  |  |  |  |
| Never married | 15/98 (15.3) | Ref | Ref | Ref | Ref | 4/23 (17.4) | Ref | Ref | Ref | Ref |
| Currently married | 24/131 (18.3) | 1.20 (0.66 – 2.16) | 0.551 | 1.08 (0.55 - 2.12) | 0.819 | 23/77 (29.9) | 1.72 (0.66 – 4.47) | 0.268 | 1.56 (0.60 - 4.06) | 0.362 |
| Previously married | 32/146 (21.9) | 1.43 (0.82 – 2.50) | 0.207 | 1.30 (0.68 - 2.51) | 0.429 | 20/69 (29.0) | 1.67 (0.63 – 4.38) | 0.301 | 1.52 (0.56 - 4.12) | 0.411 |
| **Number of sexual partners in the past year** |  |  |  |  |  |  |  |  |  |  |
| 0/1 | 10/66 (15.2) | Ref | Ref | Ref | Ref | 19/93 (20.4) | Ref | Ref | Ref | Ref |
| 2 | 13/80 (16.3) | 1.07 (0.50 – 2.29) | 0.856 | 1.05 (0.48 - 2.26) | 0.906 | 15/48 (31.3) | 1.53 (0.85 – 2.74) | 0.152 | 1.59 (0.89 - 2.85) | 0.115 |
| >=3 | 48/229 (21.0) | 1.38 (0.74 – 2.58) | 0.309 | 1.38 (0.72 - 2.62) | 0.332 | 13/28 (46.4) | **2.27 (1.29 – 4.00)** | **0.005** | **2.17 (1.22 - 3.88)** | **0.009** |
| **Perceived HIV risk** |  |  |  |  |  |  |  |  |  |  |
| Very likely | 36/169 (21.3) | Ref | Ref | Ref | Ref | 33/93 (35.5) | Ref | Ref | Ref | Ref |
| Somewhat likely | 23/137 (16.8) | 0.79 (0.49 – 1.26) | 0.324 | 0.81 (0.50 - 1.30) | 0.376 | 12/59 (20.3) | **0.57 (0.32 – 1.02)** | **0.058** | **0.55 (0.31 - 0.98)** | **0.042** |
| Unlikely | 8/41 (19.5) | 0.92 (0.46 – 1.82) | 0.802 | 0.99 (0.50 - 1.93) | 0.968 | 1/9 (11.1) | 0.31 (0.05 – 2.04) | 0.225 | 0.37 (0.06 - 2.51) | 0.311 |
| Not at all/Don't know | 4/28 (14.3) | 0.67 (0.26 – 1.74) | 0.412 | 0.74 (0.27 - 2.04) | 0.565 | 1/8 (12.5) | 0.35 (0.05 – 2.26) | 0.271 | 0.37 (0.06 - 2.39) | 0.295 |
| **Intimate partner violence** |  |  |  |  |  |  |  |  |  |  |
| No | 53/288 (18.4) | Ref | Ref | Ref | Ref | 32/115 (27.8) | Ref | Ref | Ref | Ref |
| Yes | 18/82 (22.0) | 1.19 (0.74 – 1.92) | 0.468 | 1.24 (0.76 - 2.00) | 0.388 | 15/50 (30.0) | 1.08 (0.64 – 1.81) | 0.776 | 1.15 (0.68 - 1.93) | 0.602 |
| **Most recent HIV test** |  |  |  |  |  |  |  |  |  |  |
| < 1 year | 63/282 (22.3) | Ref | Ref | Ref | Ref | 46/144 (31.9) | Ref | Ref | Ref | Ref |
| >=1 year | 8/93 (8.6) | **0.39 (0.19 – 0.77)** | **0.007** | **0.38 (0.19 - 0.77)** | **0.007** | 1/25 (4.0) | **0.13 (0.02 – 0.87)** | **0.036** | **0.13 (0.02 - 0.96)** | **0.045** |
| **Current FP use**** |  |  |  |  |  |  |  |  |  |  |
| No | 49/267 (18.4) | Ref | Ref | Ref | Ref | 21/91 (23.1) | Ref | Ref | Ref | Ref |
| Yes | 22/108 (20.4) | 1.11 (0.71 – 1.74) | 0.650 | 1.09 (0.68 - 1.72) | 0.726 | 21/65 (32.3) | 1.4 (0.84 – 2.35) | 0.201 | 1.33 (0.78 - 2.26) | 0.300 |
| **Transactional sex***** |  |  |  |  |  |  |  |  |  |  |
| No | 42/185 (22.7) | Ref | Ref | Ref | Ref | 15/81 (18.5) | Ref | Ref | Ref | Ref |
| Yes | 29/187 (15.5) | 0.68 (0.45 – 1.05) | 0.081 | 0.65 (0.43 - 1.00) | **0.048** | 32/87 (36.8) | **1.99 (1.16 – 3.39)** | **0.012** | **1.97 (1.16 - 3.34)** | **0.012** |

PR=prevalence ratio; CI=confidence interval; FP=family planning

*Participant in-migrated to community since prior survey (~18-month survey interval between RCCS survey round 19 [current round] and RCCS survey round 18 [prior round])

**Self-reported use of at least one family planning method at the time of the survey

***Sexual exploitation for respondents under 18 years of age.
