# Supplementary material for: Pre-exposure prophylaxis (PrEP) awareness, use, and discontinuation among Lake Victoria fisherfolk in Uganda: A cross-sectional population-based study
Source: PLOS Glob Public Health. 2025 May 9;5(5):e0003994. doi: 10.1371/journal.pgph.0003994 (PMC12063894; doi:10.1371/journal.pgph.0003994)
Supplement: S2 Table — (DOCX) [file pgph.0003994.s003.docx]

### **S3 Supplemental Table:** Individual-level correlates of PrEP discontinuation among female and male RCCS participants reporting PrEP ever use (n=197) in a Lake Victoria fishing community in southcentral Uganda in 2019.

|  | | **Total participants (n = 197)** | | | | |
| --- | --- | --- | --- | --- | --- | --- |
| **Characteristics** | **No. Reporting/Total (%)** | | **Unadjusted PR (95%CI)** | **P-value** | **Age-adjusted PR (95%CI)** | **P-value** |
| **Age group (years)** |  | |  |  |  |  |
| 15-19 | 4/13 (30.8) | | 0.59 (0.25 – 1.39) | 0.228 | 0.59 (0.25 - 1.39) | 0.228 |
| 20-24 | 22/42 (52.4) | | Ref | Ref | Ref | Ref |
| 25-29 | 27/46 (58.7) | | 1.12 (0.77 – 1.63) | 0.554 | 1.12 (0.77 - 1.63) | 0.554 |
| 30-34 | 20/37 (54.1) | | 1.03 (0.68 – 1.56) | 0.882 | 1.03 (0.68 - 1.56) | 0.882 |
| 35-39 | 11/27 (40.7) | | 0.78 (0.45 – 1.33) | 0.360 | 0.78 (0.45 - 1.33) | 0.360 |
| 40-44 | 7/26 (26.9) | | 0.51 (0.26 – 1.03) | 0.061 | 0.51 (0.26 - 1.03) | 0.061 |
| 45-49 | 3/6 (50.0) | | 0.95 (0.41 – 2.23) | 0.915 | 0.95 (0.41 - 2.23) | 0.915 |
| **Sex** |  | |  |  |  |  |
| Male | 56/116 (48.3) | | Ref | Ref | Ref | Ref |
| Female | 38/81 (46.9) | | 0.97 (0.72 – 1.31) | 0.851 | 0.94 (0.69 - 1.27) | 0.687 |
| **Educational Status** |  | |  |  |  |  |
| None | 7/15 (46.7) | | 1.11 (0.63 – 1.99) | 0.712 | 1.19 (0.68 - 2.08) | 0.538 |
| Primary | 54/129 (41.9) | | Ref | Ref | Ref | Ref |
| Secondary/Tertiary | 33/53 (62.3) | | **1.49 (1.11 – 1.99)** | **0.008** | **1.37 (1.02 - 1.84)** | **0.038** |
| **Recent in-migrant*** |  | |  |  |  |  |
| No | 72/155 (46.5) | | Ref | Ref | Ref | Ref |
| Yes | 22/42 (52.4) | | 1.13 (0.81 – 1.58) | 0.481 | 1.07 (0.77 - 1.49) | 0.674 |
| **Marital status** |  | |  |  |  |  |
| Never married | 8/24 (33.3) | | Ref | Ref | Ref | Ref |
| Currently married | 51/109 (46.8) | | 1.40 (0.77 – 2.56) | 0.268 | 1.47 (0.79 - 2.75) | 0.223 |
| Previously married | 35/64 (54.7) | | 1.64 (0.89 – 3.01) | 0.111 | 1.76 (0.94 - 3.28) | 0.077 |
| **Number of sexual partners in last year** |  | |  |  |  |  |
| 0 or 1 | 39/88 (44.3) | | Ref | Ref | Ref | Ref |
| 2 | 28/46 (60.9) | | 1.37 (0.99 – 1.91) | 0.059 | 1.36 (0.98 - 1.90) | 0.068 |
| >=3 | 27/63 (42.9) | | 0.97 (0.67 – 1.40) | 0.859 | 0.96 (0.66 - 1.38) | 0.813 |
| **Perceived HIV risk** |  | |  |  |  |  |
| Very likely | 57/118 (48.3) | | Ref | Ref | Ref | Ref |
| Somewhat likely | 29/55 (52.7) | | 1.09 (0.80 – 1.49) | 0.582 | 1.07 (0.79 - 1.45) | 0.667 |
| Unlikely | 4/14 (28.6) | | 0.59 (0.25 – 1.38) | 0.225 | 0.60 (0.25 - 1.43) | 0.251 |
| Not at all/Don't know | 4/10 (40.0) | | 0.83 (0.38 – 1.81) | 0.636 | 0.88 (0.41 - 1.90) | 0.750 |
| **Intimate partner violence** |  | |  |  |  |  |
| No | 61/135 (45.2) | | Ref | Ref | Ref | Ref |
| Yes | 33/61 (54.1) | | 1.20 (0.89 – 1.61) | 0.234 | 1.17 (0.86 - 1.59) | 0.308 |
| **Substantial HIV risk/likely PrEP eligible** |  | |  |  |  |  |
| No | 35/79 (44.3) | | Ref | Ref | Ref | Ref |
| Yes | 59/118 (50.0) | | 1.13 (0.83 – 1.53) | 0.439 | 1.11 (0.82 - 1.50) | 0.496 |
| **Most recent HIV test** |  | |  |  |  |  |
| < 1 year | 85/181 (47) | | Ref | Ref | Ref | Ref |
| >=1 year | 9/16 (56.3) | | 1.20 (0.76 – 1.90) | 0.441 | 1.20 (0.76 - 1.89) | 0.443 |
| **Current FP use**** |  | |  |  |  |  |
| No | 49/109 (45) | | Ref | Ref | Ref | Ref |
| Yes | 38/78 (48.7) | | 1.08 (0.80 – 1.47) | 0.609 | 1.07 (0.79 - 1.46) | 0.652 |
| **Transactional sex in last year***** |  | |  |  |  |  |
| No | 67/136 (49.3) | | Ref | Ref | Ref | Ref |
| Yes | 27/61 (44.3) | | 0.90 (0.65 – 1.25) | 0.524 | 0.94 (0.68 - 1.30) | 0.700 |

PR=prevalence ratio; CI=confidence interval; FP=family planning

*Participant in-migrated to community since prior survey (~18-month survey interval between RCCS survey round 19 [current round] and RCCS survey round 18 [prior round])

**Self-reported use of at least one family planning method at the time of the survey

***Sexual exploitation for respondents under 18 years of age
