# Supplementary material for: Pre-exposure prophylaxis (PrEP) awareness, use, and discontinuation among Lake Victoria fisherfolk in Uganda: A cross-sectional population-based study
Source: PLOS Glob Public Health. 2025 May 9;5(5):e0003994. doi: 10.1371/journal.pgph.0003994 (PMC12063894; doi:10.1371/journal.pgph.0003994)
Supplement: Attachment — Submitted filename: Response to reviewers_2024-11-11.docx [file pgph.0003994.s005.docx]

**Reviewer #1**

1) **The authors explored an important topic of public health importance. The findings will be valuable in implementing HIV preventions interventions. The manuscript can be improved by addressing the following issues:

   The tense used in the manuscript should be uniform. There is a mixture of past and past perfect tense under the description of the study population and setting.

   There is also an extensive use of abbreviations in the manuscript which makes it difficult to comprehend. My suggestion is that the abbreviations should be reduced.**

Thank you for your positive feedback on our manuscript and for recognizing the importance of our findings for HIV prevention interventions. We appreciate your constructive suggestions to enhance the clarity and readability of our work. The methods section has been reviewed for readability, with tenses corrected where necessary, and all abbreviations are now defined at their first mention.

2) **What was the actual sample size used in this study, 2,701 or 1,401??**

Thank you for your question. Of the 2,701 survey participants, 1,401 with a negative HIV test were included in the this analysis, representing the sample size of our study.

3) **Tables need to be re-designed to look simple and easy to visualize.**

The tables have been reformatted for easy visualization.

**Reviewer #2**

1) **The numbers and percentages of ever use of PrEP is not matching in line 16 and 17**

Thank you for pointing this out. We have checked the numbers and percentages, and they do indeed match. The denominators have been included for improved clarity and better interpretation of the results.

2) **Since the community is already sensitized about the programme there is awareness on PrEP in the community. However, the "knowledge" regarding PrEP will be a broader term to assess using one single question. Knowledge and awareness are interchangeably used in the discussion- I think same term throughout would be better.**

Thank you for your detailed review and valuable suggestions. We agree with the reviewer that the term *knowledge* is too broad to assess with a single question. In response, we have replaced *PrEP knowledge* with *PrEP awareness* throughout the manuscript to enhance clarity and precision.

3) **Table 2 and Table 3 are overcrowded with numbers. The number of categories in some of the variables like occupation could be reduced by clubbing.**

Thank you for your helpful suggestions regarding the tables and regression analysis. To improve readability, we have reformatted Tables 2 and 3 to reduce congestion. In addition, the categories for occupation have been reduced (there are now four instead of the previous seven): Agrarian, Fishing, Housework, and Other. “Other” comprises Boda/automobile, Trader/shopkeeper, Other, and Waitress/bartender.

4) **Regression analysis could be limited to only those variables that are significantly associated with the outcome variable.**

Thank you for the opportunity to clarify our regression analysis approach. In our analysis, we aimed to compare levels of PrEP awareness and use across different participant demographic and behavioral sub-groups to pinpoint specific populations with low awareness. In the multivariable model, we included only age as an adjustment variable. Our intent was not to construct a causal model but to highlight subgroups with particularly low PrEP awareness and use. This approach aligns with our objective to inform targeted interventions within these populations.
